# Commentary: Initial characterization of novel beaked whale morbillivirus in Hawaiian cetaceans

**DOI:** 10.3389/fmicb.2016.01205

**Published:** 2016-08-04

**Authors:** Giovanni Di Guardo, Antonio Fernández, Sandro Mazzariol

**Affiliations:** ^1^Faculty of Veterinary Medicine, University of TeramoTeramo, Italy; ^2^Institute for Animal Health, Veterinary School, University of Las Palmas de Gran CanariaLas Palmas, Spain; ^3^Department of Comparative Biomedicine and Food Science, University of PaduaPadova, Italy

**Keywords:** *Dolphin Morbillivirus*, *Cetacean Morbillivirus*, infection, cetaceans, cetacean strandings, Mediterranean Sea

The *Dolphin Morbillivirus* (DMV) epidemic which occurred between 1990 and 1992 was particularly dramatic for Western Mediterranean striped dolphins (*Stenella coeruleoalba*). This was followed by a second outbreak that simultaneously involved, between 2006 and 2008, long-finned pilot whales (*Globicephala melas*) and striped dolphins from the same area. Two additional “unusual mortality events” (UMEs), mostly affecting the Western Mediterranean striped dolphins' population, were reported in 2010–2011 along the Spanish coastline and during the first half of 2013 along the Tyrrhenian coast of Italy, respectively (Van Bressem et al., 2014). The aforementioned scenario is made even more alarming by the four DMV- and *Porpoise Morbillivirus* (PMV)-related UMEs which mainly affected, between 1982 and 2014, wild bottlenose dolphins (*Tursiops truncatus*) from the USA and Mexico (1994 UME) Atlantic seaboards (Van Bressem et al., 2014).

With respect to these virus outbreaks, we believe some issues deserve special consideration.

First of all, the numerical increase of DMV-susceptible cetacean species which has occurred in the last 5 years in the Mediterranean is a matter of concern. Indeed, evidence of DMV infection in association with either clustered or mass mortality has been recently described in fin whales (*Balaenoptera physalus*) and sperm whales (*Physeter macrocephalus*) stranded along the Italian coastline (Centelleghe et al., 2016; Mazzariol et al., 2016). Still noteworthy, a case of lethal DMV infection was previously reported in a common seal (*Phoca vitulina*) hosted by an aquatic park near Rome (Mazzariol et al., 2013).

Interestingly, a new *Cetacean Morbillivirus* (CeMV) strain, named *Beaked Whale Morbillivirus* (BWMV) and quite distantly related to DMV, has been recently characterized from 15 individuals belonging to 12 different cetacean species stranded in Hawaii between 1997 and 2014 (Jacob et al., 2016). Previous work by the same group had already shown evidence of morbilliviral infection in a Longman's beaked whale (*Indopacetus pacificus*), in a Blainville's beaked whale (*Mesoplodon densirostris*) and in a newborn, *Brucella* spp. and *Morbillivirus*-coinfected, sperm whale from Hawaiian Islands (West et al., 2013, 2015).

Of major concern are also the documented cases of DMV infection in a fin whale newborn and in a sperm whale *fetus* (Centelleghe et al., 2016; Mazzariol et al., 2016). The latter specimen, whose mother was also DMV-infected, was part of a DMV-infected, female sperm whales' pod (Centelleghe et al., 2016). To our knowledge, this should be one of the few documented cases of DMV involvement in a cetacean mass stranding and the only reported example of DMV involvement in a sperm whales' mass stranding worldwide.

Of additional concern is also the fact that CeMV, apart from considerably expanding the range of its susceptible host species both in the Mediterranean (Centelleghe et al., 2016; Mazzariol et al., 2016) and along the USA Atlantic coast (Van Bressem et al., 2014), has given rise with an apparently increased frequency, in recent years, to maternally acquired infections (West et al., 2015; Centelleghe et al., 2016; Jacob et al., 2016; Mazzariol et al., 2016). This potential “infection strategy” could provide the virus with a stronger pathogenicity, which could be further enhanced by the immune suppression “physiologically” occurring during pregnancy (Sykes et al., 2012). In this respect, the recently described biomolecular and immunohistochemical evidence of DMV in the thymus of a newborn fin whale (Mazzariol et al., 2016) provides additional support to the aforementioned hypothesis, given that an “immunotolerance-like condition” against the virus presumably developed in this whale (Weissman, 2016).

The changing “behavior” of DMV toward its “traditional” and its “new” hosts could be explained, alongside with the increasingly reported “brain-only” forms of infection among Mediterranean striped dolphins (Van Bressem et al., 2014), both by an “antiviral population's immunity enhancement” resulting from prolonged DMV exposure (Profeta et al., 2015) and by the “evolving lesions' pattern/phenotype.” As a matter of fact, the lesions observed in DMV-infected animals from the 2010 to 2011 and the 2013 UMEs in the Western Mediterranean were different from those “classically” reported in DMV-infected cetaceans (Van Bressem et al., 2014), similarly to what found in the aforementioned pod of DMV-infected, female sperm whales.

In conclusion, further research on the challenging topic of morbilliviral infections in cetaceans and, more in general, in aquatic mammals is warranted. To this aim, adequate attention should be also paid to the “emergence” of new *Morbillivirus* genus members, distantly related but still belonging to the CeMV clade, in free-ranging cetaceans from the Southern Hemisphere (Van Bressem et al., 2014).

## Author contributions

GD wrote the first draft of the Commentary. AF and SM performed at their turn, sharing it with GD, a critical and in-depth analysis of the literature concerning Cetacean Morbillivirus and Dolphin Morbillivirus infection(s), to which GD, AF and SM have also contributed in a substantial manner. Furthermore, AF and SM have carefully reviewed, thereby integrating it, the initial Commentary's draft, written by GD, with the last version of the manuscript having been fully agreed by the three authors.

### Conflict of interest statement

The authors declare that the research was conducted in the absence of any commercial or financial relationships that could be construed as a potential conflict of interest.
